# Factors influencing willingness and ability to pay for social health insurance in Nigeria

**DOI:** 10.1371/journal.pone.0220558

**Published:** 2019-08-02

**Authors:** Yewande Kofoworola Ogundeji, Babatunde Akomolafe, Kelechi Ohiri, Nuhu Natie Butawa

**Affiliations:** 1 Department of Health Financing, Health Strategy & Delivery Foundation (HSDF), Abuja, Nigeria; 2 Department of Planning Research & Statistics, Ministry of Health, Kaduna, Nigeria; University of Florida, UNITED STATES

## Abstract

**Background:**

Many low and middle-income countries are increasingly cognisant of the need to offer financial protection to its citizens through pre-payment schemes in order to curb high out of pocket expenditure and catastrophic spending on healthcare. However, there is limited rigorous contextual evidence to make decisions regarding optimal design of such schemes. This study assesses the willingness-to-pay (WTP) for the recently introduced state contributory health insurance scheme (SHIS) in Nigeria.

**Methods:**

The study took place in 6 local government areas in Kaduna state, North-west Nigeria. Data were collected from a household survey using a three-stage cluster sampling approach, with each household having the same probability of being selected. Interviews were conducted with 4000 individuals in 1020 households. Contingent valuation was used to elicit the willing to pay (WTP) for the household using the bidding game technique. The relationship between socioeconomic status and WTP was also examined using logistic regression models.

**Findings:**

About 82% of the household heads were willing to pay insurance premiums for their households, which came to an average of 513 Naira (1.68 USD) per month per person. The average amount individuals were willing to pay was lower in rural areas (611 Naira) compared to urban areas (463 Naira). These results were influenced by household size, level of education, occupation and household income. In addition, only 65% of the households had the ability to pay the average premium.

**Conclusion:**

Socioeconomic factors influence individuals’ WTP for contributory health insurance schemes. It is important to create awareness about the benefits of the insurance scheme, especially in rural areas, and in both the formal and informal sectors in Nigeria. WTP information can inform the amount of insurance premiums. However, it is important to consider differences between the WTP and the cost of benefits package to be offered, as the premium amount may need to be subsidized with public financing.

## Introduction

In recent years, equitable access to quality healthcare towards universal health coverage (UHC) has become priority in many low and middle-income countries. Financial protection, which has to do with how much people have to pay out of pocket is an integral component of achieving universal health coverage [1]. There is substantial evidence that show that reliance on out-of-pocket payments (OOP) as the main payment source for healthcare does not only have an adverse effect on demand for services, but increases the financial burden of households leading to impoverishment [2–4]. Additionally, available evidence indicates that per capita spending on health in many low and middle income countries is likely to increase rapidly the long run [5–7].

In Nigeria, OOP contributes over 70% of the total health expenditure, greatly exceeding the recommended 30% threshold [8,9]. Nigeria’s OOP is among the highest in the world and the highest in Africa, which translates to catastrophic spending in a majority of households [10].

Nigeria has shown some commitment to reducing OOP and increasing access to quality basic health services through the National Health Act signed into law in 2014. The Act provides a legal framework for the provision of health services and establishes an organisational and management structure for the health system in Nigeria. To achieve this important objective of providing quality healthcare services to all Nigerians, “the Act specifies that all Nigerians shall be entitled to a Basic Minimum Package of Health Services (BMPHS) to be funded by a basic health care provision fund (BHCPF) which would be derived from contributions of not less than one percent (1%) of the Consolidated Revenue Fund (CRF) of the Federal Government of Nigeria” [11]. According to the developed guidelines for disbursement of the BHCPF, 50% of the BHCPF is expected to go towards the expansion and funding of BMPHS which States could leverage on by establishing a State contributory health insurance scheme. The potential and prospects of the BHCPF have caused many States in Nigeria (including Kaduna, Lagos, and Delta) to set into motion the design and implementation of a State Social Health Insurance Scheme (SHIS).

Nigeria has experimented with forms of social health insurance schemes in the past. For example, the National Health Insurance Scheme introduced in the year 2000, with coverage of only about 4% of the population, a majority of whom are federal civil service employees [12]. The limited coverage of this scheme has often been attributed to the lack of acceptability and unwillingness to pay premiums, especially within the informal sector. This is consistent with evidence from other countries that suggest that achieving financial sustainability and effective cross-subsidisation through high enrolment rates especially within the informal sector are critical success factors in implementing SHIS [13,14].

Given that the informal sector makes up of about 70% of Nigeria’s workforce, it is important to assess the viability of the scheme’s funding through premium payments by exploring the readiness of the population to enrol in the proposed scheme and pay premiums. An approach to this is a willingness to pay (WTP) study. Literature, however, suggest that respondents self-reported WTP have not always translated into payment because of several factors that affect ability to pay such as size of household income, gender etc. [15–19]. As a result, WTP studies provide more robust evidence with better value when ability to pay (ATP) and other potential determinants are assessed.

In line with the National direction to implement decisions and policies based on evidence, a WTP study will provide evidence on the potential coverage of SHIS, the potential amount individuals are willing to pay for premiums, and factors which influence it WTP, which would inform mobilisation and sensitisation strategies especially in creating awareness and increasing enrolment rates. The study will also provide context-specific insight into the potential envelope which is pertinent to the design of the benefit packages offered to enrollees under the scheme to ensure the sustainability and effectiveness of the scheme.

Using a case study of social health insurance scheme in Kaduna state, this study sought to:

1. Assess willingness to pay (WTP) for a state social health insurance scheme in Kaduna state
2. Explore the factors that are likely to influence household willingness to participate in the health insurance scheme in Kaduna state
3. Assess the ability to pay (ATP) for insurance premiums in Kaduna state

## Methods

### Study setting, sampling, and data collection

The study utilised a cross-sectional household survey in Kaduna State of Nigeria. Adults over 18 years were interviewed to collect information on demographics, assets, wealth, household expenditures, health care utilisation, health care expenses and willingness to pay for healthcare, and financial protection (S1 File) details the survey questionnaire).

A stratified cluster design was adopted for this survey. The first stage was a stratified random sampling of LGAs within senatorial districts. Kaduna State is divided into three senatorial districts (Kaduna south, Kaduna central, Kaduna North), which contain 23 local government areas (LGAs), further subdivided into census enumeration areas (EAs). In the second stage, the sampled LGAs was stratified into urban and rural areas and EAs were selected using probability proportionate to size from each stratum of the selected LGAs. In the third stage, a fixed number of households were selected in every urban and rural cluster through random sampling based on the household list. A total number of 17 enumeration areas were covered in each sampled LGA. 10 households were selected per EA. 1,020 households were canvassed in the 6 selected LGAs of Kaduna State (Table 1).

**Table 1:** Sample allocation of clusters and households by residence for Kaduna state.

Sample Selection	Kaduna State		
	Urban	Rural	Total
EA/Clusters	21	81	102
Households	210	810	1020

Interviews were conducted at the respondent’s home. The head of household and men or women above 18 years present in the household on the night before the survey were eligible to be interviewed for the survey. Data collection period spanned over 6 weeks and incorporated the use of trained enumerators and supervisors.

### Ethical approval

This study was approved by the Kaduna State Health Research Ethics Committee. Informed consent was given verbally.

### Eliciting willingness to pay

Contingent valuation was used to elicit the WTP for household head, and other members of the household using the bidding game technique. Three iterations were used in the bidding game depending on their response to the starting bid question. The final bid question was an open-ended amount that indicated the respondents’ maximum WTP. A brief introductory explanation and scenario about health insurance were provided to the respondents before determining their levels of WTP for the scheme. The concept of health insurance and its attributes were explained before starting the bidding game. The starting point bid of 1000 naira was based on average premium contributions of existing voluntary schemes in Nigeria such as the voluntary contributor's social health insurance programme in Nigeria (VCSHIS).

The bidding game iteration for eliciting WTP for the individual were:

1. If the price of a monthly insurance premium per person is 1000 Naira, will you be willing to pay?
2. What if the premium is 700 Naira, will you be willing to pay?
3. What if the premium is 450 Naira, will you be willing to pay?
4. What is the maximum amount you are willing to pay for health insurance scheme?

### Economic model used to explore the determinants of willingness to pay

We used two models to explore determinants of WTP. First, we used a logistic multivariate regression model to explore the relationship between socioeconomic factors and whether individuals were willing to pay for health insurance. We also utilised a linear multivariate regression model to explore the determinants of the relative amount respondents were willing to pay.

Table 2 describes the dependent and independent variables used in the logistic regression model.

**Table 2:** Description of the dependent and independent variable hypothesised to explain willingness to pay.

Variables	Description	Measurement
**WTP (dependent variable)**	If respondents are willing to pay for health insurance or not	0 = No 1 = Yes
**Income**	The monthly earning of the head of the household	0 = Low income 1 = High income
**Sex**	Whether male or female	0 = Female 1 = Male
**Education**	The highest level of education attained	0 = Low education level 1 = High education level
**Household size**	Total number of residents in the household	0 = Low household size 1 = High household size
**Geographic location**	Whether respondent is residence in rural or urban location	0 = Rural 1 = Urban

Numerical variables such as income and household size were converted to dummy binary variables using the median value to set thresholds for the categories. A sensitivity test was conducted by setting different thresholds for level of education and income to test the corresponding effect on WTP. In the linear regression model, the dependent variable was the amount the respondents were willing to pay, whilst independent variables were socioeconomic variables explored in the logistic regression model. All analyses were conducted on SPSS Statistics version 17.0.

### Ability to pay

The expenditure-income ratio approach was used to estimate the ability of the respondent to pay the WTP amount. We utilised a 5% health expenditure-income ratio based on evidence from developing countries that suggest households that do not experience impoverishment due to healthcare costs spend less than 5% of monthly income on health [20,21].

## Results

### Socio-economic and demographic characteristics

Table 3 outlines the socio-economic and demographic characteristics of the respondents. A total of 985 respondents were interviewed in 6 LGAs in Kaduna State with 72.5% of the respondents from rural areas while the remaining 27.4% were from urban areas.

**Table 3:** Key socio-economic characteristics of respondents.

	Rural		Urban		Total	
Income (naira)	Frequency	%	Frequency	%	Frequency	%
**0–19999**	342	52.7	90	26.8	432	43.9
**20000–39999**	173	26.7	92	27.4	265	26.9
**40000–59999**	84	12.9	74	22.0	158	16.0
**60000–79999**	22	3.4	27	8.0	49	5.0
**80000–99999**	9	1.4	17	5.1	26	2.6
**100000–119999**	6	0.9	16	4.8	22	2.2
**120000+**	13	2.0	20	6.0	33	3.4
**Age**						
**20–29**	57	8.8	32	9.5	89	9.0
**30–39**	184	28.4	110	32.7	294	29.8
**40–49**	195	30.0	92	27.4	287	29.1
**50–59**	126	19.4	65	19.3	191	19.4
**60+**	87	13.4	37	11.0	124	12.6
**Sex**						
**Female**	41	6.3	28	8.3	69	7.0
**Male**	608	93.7	308	91.7	916	93.0
**Occupation**						
**Govt. worker**	91	14.0	60	17.9	151	15.3
**Organized private**	10	1.5	22	6.5	32	3.2
**Pensioner**	32	4.9	17	5.1	49	5.0
**Self-employed**	399	61.5	194	57.7	593	60.2
**Student**	10	1.5	8	2.4	18	1.8
**Unemployed**	23	3.5	12	3.6	35	3.6
**Others**	84	12.9	23	6.8	107	10.9
**Education**						
**Primary comp**	98	15.1	27	8.0	125	12.7
**Post graduate**	15	2.3	19	5.7	34	3.5
**Secondary**	236	36.4	117	34.8	353	35.8
**University**	78	12.0	101	30.1	179	18.2
**(blank)**	157	24.2	48	14.3	205	20.8
**Others**	63	9.7	24	7.1	87	8.8
**No education**	2	0.3	0	0.0	2	0.2

### Willingness to pay for contributory health insurance scheme

Of the total number of respondents, 82% were willing to pay health insurance premiums. The mean amount respondents were willing to pay is N513 ± N47 ($1.68) per month, with urban respondents willing to pay N611 ± N63 ($2), whilst respondents in rural areas were willing to pay N463 ± N62 ($1.51) naira per month (Table 4). Male respondents and unmarried respondents were willing to pay a higher amount compared to female respondents and married respondents respectively. An indication of respondents' willingness to join the insurance scheme was highest at (30.4%) among age group within 30–39 years and least within the age group of 20–29 years (9.4%).

**Table 4:** Descriptive statistics for amount respondents were willing to pay.

	Male	Female	Married	Single	Urban	Rural
**Mean**	519	426	511	783	611	464
**SE**	25	54	26	130	32	32
**Median**	450	350	450	500	500	300
**Mode**	500	500	500	500	500	200
**SD**	692	391	692	700	527	736
**CI (95.0%)**	49	108	51	266	63	63

### Factors that influence willingness to pay

Table 5 shows the relationship between the dependent and independent variables in our regression model. The results showed a statistically significant positive relationship 2.295(p = 0.001) between household income and WTP. More specifically, as income increases, the likelihood to pay for health insurance premium increases.

**Table 5:** Result of logistics multivariate regression analysis.

Independent Variables	Multivariate Regression		Univariate Regression	
	Odds Ratio	P-value(95% CI	Odds Ratio	P-value(95% CI
**Income**	2.295	0.001	1.633	0.029
**Sex**	1.135	0.802	1.144	0.786
**Education**	0.419	0.012	0.484	0.005
**Occupation**	0.723	0.367	0.533	0.024
**Household size**	0.668	0.089	0.679	0.090
**Geographic Location**	1.118	0.651	1.193	0.436

We also found a negative statistically significant relationship 0.419 (p = 0.012) between the level of education and WTP, which suggest the individuals with post-secondary education are less likely to pay for health insurance. Findings from the linear regression model further showed that the amount respondents were willing had a statistically significantly positive association with income, and occupation (Table 6). The sensitivity analyses conducted for the logistic regression model did not yield any significant differences in results (S1 Table).

**Table 6:** Result of linear multivariate regression analysis.

Independent Variables	Multivariate regression		Univariate regression	
	B coefficient	P-value(95% CI	B coefficient	P-value(95% CI
**Income**	0.134	0.032	0.182	0.000
**Education**	0.042	0.434	0.142	0.001
**Occupation**	0.114	0.031	0.174	0.000
**Household size**	-0.33	0.476	0.079	0.937
**Geographic location**	0.003	0.943	0.062	0.168
**Sex**	-0.052	0.238	-0.051	0.254

### Ability to pay

Using the health-income expenditure approach, we evaluated the affordability of the monthly insurance premium of 513 naira and found out that only 65% of the sampled households could afford to pay for the health insurance premium (Table 7).

**Table 7:** Expenditure to income approach for ability to pay.

	>5% of household income	<5% of household income
**Frequency**	282	525
**Percentage (%)**	35%	65%

## Discussion

This study explored the willingness and ability to pay for social health insurance in Kaduna (located in the north western part of Nigeria), Nigeria. The mean amount that household heads were willing to pay per month for insurance premiums was 513 ± 47 naira ($1.68) in Kaduna. Our findings differed considerably from studies conducted in the eastern region (Anambra and Enugu State) and in the north-central region (Kwara State) of Nigeria which found the amount respondents were willing to pay was 260 naira per month and 522 naira per year respectively [22,23]. This disparity is likely as a result of differences in socio-economic conditions, limited sample size, inflation (due economic differences at the time of the studies), and differences in geo-political zones. For instance, a representative sample size of 1020 households were sampled for our survey compared to the 360 households that were sampled in Ilorin South Local government which was not representative of Kwara State. Similarly, differences in macroeconomy and higher living standards could affect the amount individuals are willing to pay for health insurance.

Similar to our findings showing the disparities between amount individuals were willing to pay for health insurance premiums in rural compared to urban areas, a study in Namibia found out that individuals in urban areas were willing to pay 47.50 NAD (6.60 USD) per month for health insurance premiums [17]. Households in rural areas generally earn less income due to high dependence on subsistence farming and petty trading to meet daily financial needs, which may explain the relatively high premiums people are willing to pay in urban areas compared to rural areas. Consequently, to successfully implement SHIS, the government needs to consider the economic status of those in the rural areas and/or the poor and vulnerable who may not be able to pay high premiums or afford it at all. A way to do this is to set premium levels on a sliding scale as opposed to flat rates premiums to take into consideration the socioeconomic status of the citizens [24].

Contrary to what literature suggest, we found out that individuals who completed higher educational qualifications were less likely to be willing to pay for health insurance premiums. Many studies suggest that individuals who are more educated are more aware of the benefits of insurance coverage and therefore will be more willing to pay [22,25–27]. However, a few studies have demonstrated contrary findings similar to ours [28,29], and there are few possible explanations for this atypical trend. Individuals with higher educational qualifications may be able to better appraise options. For example, they may be able to assess service quality issues that may affect the ultimate benefits of enrolling in the scheme. In addition, there is a rising demand for pharmaceuticals for non-communicable diseases and advanced medical technologies which are typically not included in the benefit package [7,30]. Consequently, they might have issues trusting the system due to the fear of the benefit package not meeting their health demands or not getting value for money. To address this, revision and expansion of the benefit package might be necessary to cater to the needs of these individuals.

Our study further found that premiums that can be afforded by individuals in the informal sector are likely to be considerably lower, which has an implication for the SHIS fund, as over 76% of the working population of the respondents work in the informal sector. Furthermore, the dependency ratio in Kaduna is high and will potentially put more pressure on the working population which are mostly in the informal sector [31]**.** In addition, only 65% of the respondents could afford the average amount the respondents were willing to pay. It is therefore pertinent that subsidisation and variable mechanisms of premium collection from the informal sectors are explored. These could include enrolment of microfinance loan recipients through a partnership between the micro-finance bank and the insurer, piloted in Lagos state, Nigeria [32]. Onsite enrolment in market stalls and booths was also used successfully to collect premiums from the informal sector in Nicaragua [33]. The government should also explore opportunities to subsidise premiums to allow the indigenous population to enrol in the scheme without facing further financial hardship. This may include donor support and government matching subsidies on contributed premiums which is similar to the mechanism experimented with in Tanzania where the insurance scheme was partly funded by the Government, which contributed towards achieving higher coverage rates in the country [34].

An important use of this willingness to pay study is for planning, designing, and implementing the insurance scheme especially as it relates to the benefits package that will be offered to enrolees. The amount respondents are willing to pay, coupled with the respondents’ ability to pay may give an indication of the fiscal space available for the social health insurance scheme fund, which will in turn guide the range and the sustainability of the benefits package offered [35].

## Conclusion

In summary, income, size of the household, level of education and formal employment amongst other factors matter in people’s choice or willingness to pay premiums, as well as the amount they are willing to pay for contributory health insurance schemes in Nigeria. The findings of this study also buttress the fact that due to contextual differences, formative research and evidence is critical in informing successful planning, designing, and implementation of social health insurance schemes. The health insurance regulatory agencies in the States in Nigeria should put in place awareness strategies to sensitise both formally and informally employed individuals on the benefits of the insurance scheme and explore innovative mechanisms to collect premiums from individuals in the informal sector. Additionally, the State governments should carefully review affordability in setting insurance premium for different segments of the society, including considerations for the provision of insurance premium subsidies for the poor and vulnerable in short to medium term.

## Supporting information

S1 File Household survey study protocol. (DOCX)

S1 Table Sensitivity test for level of education and income. (DOCX)
