# Supplementary material for: Factors influencing willingness and ability to pay for social health insurance in Nigeria
Source: PLoS One. 2019 Aug 2;14(8):e0220558. doi: 10.1371/journal.pone.0220558 (PMC6677309; doi:10.1371/journal.pone.0220558)
Supplement: S1 File — (DOCX) [file pone.0220558.s001.docx]

**HOUSEHOLD SURVEY STUDY PROTOCOL**

Contents

[1. Background 5](#_Toc495030772)

[2. Study Objectives 6](#_Toc495030773)

[2.1 Primary Objective 6](#_Toc495030774)

[2.2 Secondary Objectives 6](#_Toc495030775)

[3. Methodology 6](#_Toc495030776)

[3.1 Study Design 6](#_Toc495030777)

[3.2 Sampling Frame 7](#_Toc495030778)

[3.3 Sampling Procedure 7](#_Toc495030779)

[3.4 Sample Size 8](#_Toc495030780)

[3.4 Selection of Respondents 8](#_Toc495030781)

[3.5 Data Collection 8](#_Toc495030782)

[3.6 Questionnaires 9](#_Toc495030783)

[3.7 Pre-test 9](#_Toc495030784)

[3.8 Recruitment and Training of Enumerators and Supervisors 10](#_Toc495030785)

[3.9 Advocacy, Sensitization and Mobilization 11](#_Toc495030786)

[3.10 Fieldwork and Monitoring 11](#_Toc495030787)

[3.11 Contingency Plan 11](#_Toc495030788)

[3.12 Data Management 12](#_Toc495030789)

[3.13 Fieldwork and Monitoring 13](#_Toc495030790)

[3.14 Data Collection 13](#_Toc495030791)

[3.15 Data Cleaning 13](#_Toc495030792)

[3.16 Data Analysis 14](#_Toc495030793)

[4. Ethical Approval and Human Subjects Considerations 14](#_Toc495030794)

[4.1 Risks 15](#_Toc495030795)

[4.2 Benefits 15](#_Toc495030796)

[4.3 Compensation 15](#_Toc495030797)

[4.4 Informed Consent 15](#_Toc495030798)

[4.5 Protection of Privacy and Confidentiality 15](#_Toc495030799)

[4.6 Adverse Events 16](#_Toc495030800)

[5. Dissemination of Results 16](#_Toc495030801)

[6. Limitations 16](#_Toc495030802)

[7. Workplan 17](#_Toc495030803)

# 1. Background

Kaduna State is the third most populous state in Nigeria, the twelfth largest state in land mass with projected population of 7.1 million in 2016. Over the years, the state government has implemented reforms to address the health system challenges and meet the aspirations of its people for improved health. The Kaduna State Government (KDSG) espouses the bold vision of, “a state where quality health care services are available, accessible and affordable to its citizens in an equitable manner and on a sustainable basis through active participation of all individuals and communities”^[[1]](#footnote-1)^. This vision has been translated into State strategic and operational plans, including the Kaduna State Medium Term Plan, (2010-2015), Kaduna State Strategic Health Development Plan (KDSSHDP 2010-2015), Integrated Management of Childhood Illnesses, the Safe Motherhood Operational Plan (2008), Routine Immunization Strategy and Operational Plan, and the FMCH Implementation Plan. Implementation of these plans have yielded modest level of progress in health financing, delivery and outcomes in the state.

Budgetary provision was the major constraint to effective implementation and achievement of the health development plans and targets respectively. More importantly, ambitious reform plans were backed by incremental budgetary support within the framework of a Medium-Term Sector Strategy (MTSS). In the absence of adequate budgetary funding of healthcare delivery, households continued to bear the greatest financial burden, which is estimated nationally at about 70 percent of total health expenditures^[[2]](#footnote-2)^. High levels of out-of-pocket (OOP) expenditure by households limit utilization of preventive care, raise the likelihood that households are forced into seeking care only when illness if fully developed, shift the distribution of healthcare disproportionately toward curative care, and ultimately reduced efficiency of health expenditure. Financial risk protection and prepayment schemes are known to support greater and more effective utilization of healthcare services and ultimately improvement in health outcomes. But coverage of such schemes remains very low even when households are willing to pay for the needed protection from catastrophic and impoverishing healthcare expenditures.

Health accounts constitute an important healthcare policy and planning tool. In addition to aiding answers to several important policy questions, it demonstrates how healthcare resources are raised and spent, on which services by which providers and through what schemes. While Health Accounts (NHA) have been estimated at the national level for the period 1998-2014, the estimates are severely limited in answering similar policy questions at the state level. It is therefore imperative for Kaduna State to develop its own estimates of health accounts.

A household survey will be conducted in Kaduna state to provide the requisite evidence for the state governments to understand the current levels of household OOP expenditures and catastrophic expenditure levels, healthcare utilization patterns and challenges, willingness-to-pay for financial risk insurance and prepayment schemes and other relevant health behaviours. These data will contribute to estimating the Sub-National Health Account (SNHA) for Kaduna state and to inform development of appropriate policies and strategies to reduce the burden of health expenditures on poor households.

# 2. Study Objectives

In order to inform the state health accounts, this study will achieve the following objectives:

### 2.1 Primary Objective

The overall objective of the study is to estimate the current levels of financial protection on health and out of pocket (OOP) expenditure which is defined as any direct outlay by households, including gratuities and in-kind payments, to health practitioners and suppliers of pharmaceuticals, therapeutic appliances, and other goods and services whose primary intent is to contribute to the restoration or enhancement of the health status of individuals or population groups^[[3]](#footnote-3)^.

### 2.2 Secondary Objectives

The household survey would further provide insights for Kaduna state in the following areas:

1. Household utilization of health services
2. Willingness to pay for healthcare financial protection
3. Catastrophic payments for health care^[[4]](#footnote-4)^

# 3. Methodology

### 3.1 Study Design

The study will utilize a cross sectional household survey in Kaduna State of Nigeria. The survey will interview adults over 18 years to collect information on demographics, asset wealth, household expenditures, health care utilization, health care expenses and willingness to pay for healthcare financial protection. The survey will follow the general sampling strategy used for the Nigeria Demographic and Health Survey (NDHS) 2013 and the Harmonized Nigerian Living Standard Surveys (HNLSS) 2009-10. The sampling strategies of both surveys were designed to produce reliable estimates for key indicators at the national level, as well as for urban and rural areas and each of the country’s six geographical zones. Based on their sample design, we will apply state-level sampling strategy and stratify by senatorial districts, urban and rural areas.

### Sampling Frame

The sampling frame that will be used for this study include:

- List of Enumeration Areas (EA) with Sketch Maps.
- Household list in each of the sampled EAs.

Kaduna state is divided into three senatorial districts (Kaduna south, Kaduna central, Kaduna North) and 23 local government areas (LGAs), which are further subdivided into census enumeration areas (EAs). Table 1 below provides the total number of LGAs and EAs for Kaduna state and their respective average number of inhabitants in an EA. A list of EAs with their corresponding maps will be sourced from National Population Commission (NPopC). After the selection of the EAs and before the main survey, a household listing operation will be carried out in all of the selected EAs/clusters.

| **Table 1: Population and Enumeration Areas** | | |
| --- | --- | --- |
| **S/N** | **Kaduna State** | **Number** |
| 1 | Local Government Areas | 23 |
| 2 | Enumeration Areas (EAs) | 21,792 |
| 3 | Average EA size (Individuals) | 280 |
| Sources: NDHS 2013, NPopC 2006 | | |

### Sampling Procedure

A three-stage sampling method will be used in the selection of the Households:

- First stage is the stratified random sampling of the Local Government Areas (LGA).
- Second stage is the selection of the Enumeration Areas using probability proportionate to size (pps).
- Third stage is the random sampling of the households.

A stratified cluster design will be adopted for this study. The first stage will be stratified random sampling of LGAs within senatorial districts and other relevant socioeconomic parameters only to avoid some contextual issues around the LGA population figures . In the second stage, the sampled LGAs will be stratified into urban and rural areas and EAs/clusters will be selected using probability proportionate to size from each stratum of the selected LGAs. A household listing will be developed for each of the selected EAs/clusters. In the third stage, a fixed number of households will be selected in every urban and rural cluster through random sampling based on the household list.

### Sample Size

- A total number of 6 LGAs will be sampled in the state across the 3 senatorial districts.
- A total number of 17 Enumeration Areas will be covered in each sampled LGA.
- 10 Households will be selected per EA.
- 1,020 households will be canvassed in the 6 selected LGAs of Kaduna state.

Table 2 below shows the sample allocation of clusters and households by residence for Kaduna state. A total of 1,020 households and 6 LGAs will be sampled in the state. Two LGAs will be sampled from each of the 3 senatorial districts in the state with 17 EA/clusters per LGA, and 10 households per EA/cluster.

| **Table 2 Sample Selection^[[5]](#footnote-5)^** | | | |
| --- | --- | --- | --- |
| **Sample Selection** | **Kaduna State** | |  |
|  | **Urban** | **Rural** | **Total** |
| EA/Clusters | 21 | 81 | 102 |
| Households | 210 | 810 | 1020 |

### 3.4 Selection of Respondents

All adults (over 18 years) will be interviewed in selected households separately with the full survey, excluding the household characteristics, willingness-to-pay, and consumption modules, which will be administered only to the household heads. Women with children will be interviewed on their own health seeking practices as well as those of their children. Adult household members will be asked about individual characteristics (e.g., age, education level), health utilization and expenditures, and health-seeking behaviours. All available eligible adults will be interviewed upon the first visit. In the case where an eligible adult is absent, we will explore the following options: i) Call back to the household to complete the outstanding household members. (ii) Use of mobile phones to interview the eligible adult, if available and allowed by the household respondent.

### 3.5 Data Collection

Interviews will be conducted at the respondent’s home at a location where he/she feels comfortable. In line with global best practice, the study instruments will be developed and reviewed by the study team to ensure that the information collected is relevant to research objectives and policy interests, thereby making the best use of the respondents’ time in the survey.

The survey is expected to last for approximately 45-60 minutes for the head of household interviews and 30 minutes for other adult household members. The survey will be administered in English with translation to Hausa in order to provide respondents with the preferred language option. Households are defined as “a person or group of person, related or unrelated, who usually live together in the same dwelling unit, have common cooking and eating arrangements, and acknowledge one adult member as the head of the household.” The head of household and men or women above 18 years present in the household on the night before the survey are eligible to be interviewed for the survey.

### 3.6 Questionnaires

There will be one household questionnaire with 11 sections administered to either all eligible members of households or the head of household only. The following modules and their relevant sections include:

- General information (State, LGA, EA name)
- All eligible household members
  - Verbal informed consent
  - Household roster/Individual background information (Name, sex, education, literacy, age, etc…)
  - Health seeking behaviour – inpatient and outpatient services
  - Individual expenditures on health – by inpatient and outpatient services
  - Access to health insurance
- Head of household only
  - Perceived quality of healthcare services
  - Willingness to pay for contributory health scheme
  - Household characteristics
  - Household consumption

Modules will be based on previously used questions from the NDHS and HNLSS and additional questions relevant to the context of the study as appropriate, . The questionnaire will be reviewed and finalized in coordination with the relevant stakeholders; designed and administered using an android application on mobile phones or tablets via an electronic platform; and synchronized with a web-based Survey CTO platform.

### 3.7 Pre-test

A pre-test of the draft questionnaire will be conducted in a small number of EAs not selected for the main survey to assess its quality. The pre-test will address the following questions:

- Are the questions and responses valid and reliable?
- Are they appropriate? Are they both necessary and sufficient?
- Do the sections of the questionnaire and the questions within sections have a logical flow?
- Are the skip instructions correct?
- Is there evidence of question order effects?

Others issue that will be addressed are: actual time to complete each questionnaire, clarity of information provided and instructions, question wording, and response categories. Problems and potential errors observed during the pre-test will be addressed and resolved by the study team prior to fieldwork.

### 3.8 Recruitment and Training of Enumerators and Supervisors

Enumerators and supervisors will be recruited from an experienced pool of outsourced staff from Kaduna State. Recruited enumerators will be knowledgeable on current population-based survey methodology and practices. Enumerators will satisfy following criteria:

- Minimum of BSc/HND in relevant Disciplines/Subjects
- Experience in population based survey
- Good communication skills
- Familiarity with the study environments
- Ability to speak local language of the study area
- Experience in the use of electronic data collection will be an added advantage.

We will recruit three teams of data collectors and three supervisors. Each team will consist of six enumerators and an assigned supervisor. Enumerators and Supervisors will participate in a four-day training program inclusive of the pilot test. The training content will include, among others: introduction to the survey, review of the questionnaires, use of Computer-Assisted Personal Interview (CAPI) device, logistics arrangements, role definition for supervisors and enumerators, understanding the survey and survey questions, interviewing techniques, ethical considerations and informed consent, data security, household selection process, and review of survey schedule and process.

The training will have the following objectives:

- Understanding data collection techniques through interviews and use of correct language, behaviour, and ethics
- Understanding the questionnaire content and purpose
- Understanding the logistics and timeframe for the survey
- Practicing and gain fluency in interviewing with the final survey questionnaire, electronic data collection and data security, and the quality improvement checklist as a supervision tool
- Gaining skills in supervision, interviewing and good survey techniques

In addition, the trainees will be given the enumeration area sample maps and survey instruction manual containing: procedures and responsibilities for enumerators and supervisors, examples of problems, causes and remedial actions, quality improvement checklist and daily interview form.

### 3.9 Advocacy, Sensitization and Mobilization

Communities will be adequately informed to improve their understanding of essence, relevance and benefit of the survey to the project environment. We will coordinate with departments in selected LGAs that are responsible for the coordination of traditional leaders and community based organizations.

### 3.10 Fieldwork and Monitoring

Fieldwork is expected to last for twenty-three days. All communities within selected clusters will be informed in advance of the survey and random selection of households for interview through communication with community leaders. Quality checks of instruments will be programmed prior to the start of fieldwork and monitored throughout. All data will be submitted to the central server at least once daily. There will be three main levels of data quality assurance during data collection:

1) Quality checks will be programmed into the electronic data collection software to ensure that data have been entered for all questions and skip patterns are correct;

2) The team supervisor is responsible for reviewing all completed surveys by his/her team daily;

3) Transmitted data will be verified daily by the central server supervisor. Areas prone to error will be identified for immediate action at each level for missing or outlying values. Data quality control measures during fieldwork are detailed further below.

### 3.11 Contingency Plan

***Absenteeism:*** More than the required enumerators (6 reserve enumerators) will be trained to ensure the best enumerators for the job. Trained substitutes will be available if a team member is sick or needs to leave for any reason.

***Bad weathers/Unavailable*** *Respondent/Call back:* Enumerators will be prepared to conduct the survey at unconventional times as dictated by weather to meet the needs of the survey populations - in case of bad weather (e.g. during late evening or very early morning). Also, the enumerators will be equipped with kits such as umbrella, raincoats, etc.

***Security:*** If a selected EA is not accessible due to security challenges, a replacement EA will be selected.

### 3.12 Data Management

The data will be collected electronically with the use of a structured questionnaire which will be preloaded onto a smart phone and sent to a central server - Survey CTO web platform, a renowned US-based web server that is secured and encrypted. The electronic platform will have the ability to collect data offline or online with the universal web interface and can submit data securely to the central server via multiple layers of encryption and data redundancy. The electronic versions of the questionnaire will be tested by the programmer and study team prior to data collection training and pretesting to ensure they are working correctly, include appropriate skip logic, required fields, and match the numbering and coding of the paper survey to the extent possible. All smart phones installed with the software and questionnaire will be password protected and all entered data will be encrypted. The data will be verified daily by a supervisor in charge of a team who checks for data consistency and validity.

If possible, each device will have a SIM card and will send data to a secure, web-based server via the mobile data network. Data will be uploaded nightly, network permitting. Otherwise, data may be downloaded onto (password protected and encrypted) field laptops at regular intervals. Teams will travel to the field with mobile hotspots and extra devices in case of problems with syncing devices directly to the server. In addition, extra power supply will be carried to the field in case electricity is unavailable. Data will remain on the device until the Data Manager has verified that it has been uploaded onto the server. Each device will be assigned to a specific data collector to help ensure that in case of queries, we are certain as to who conducted the interview.

After the first two days of data collection, and periodically throughout data collection, a study team members that are granted privileges to the server will review the uploaded data to check for errors, and other essential variables. Any user- errors that need to be corrected at this point will be communicated to the field teams. If any programming errors are identified, a new version of the electronic survey may be prepared, tested by the study analyst, and then re-uploaded onto all the devices. Field supervisors will verify that all data collected on the old form have been uploaded and removed from devices. They will then verify that all devices have the new form before deleting the old form. The field supervisors will prepare a memo to the study team member indicating the names of each device and the date on which each device was checked. Depending on the severity of the necessary changes, the data analyst will decide whether to keep the old data for merging with the new, or not using the old data and this decision will be documented.

Following the transmission of all interviews to the central server, consistency checks and other quality assurance measures that are built into the data entry program will be automated, and overall data cleaning through the process of running frequencies of key indicators/variables will be carried out by a study team member at the Abuja office.

### 3.13 Fieldwork and Monitoring

Quality control of data will be employed at various stages, during data collection, data cleaning and data assessment. This will be emphasized during training to check internal and logical consistencies which include:

### 3.14 Data Collection

***Selection of the households:*** The households will be pre-selected at the central level of each enumeration area prior to the start of fieldwork rather than by enumerators in the field who may have cultural or social pressures to bias the selection process.

***Coding of all instruments with a unique serial number:*** Each instrument will be assigned a unique number to de-identify personal information which will be represented by their state code, EA code and respondent identification number to avoid duplication at the central level. All households and household respondents will also be assigned unique identification numbers.

***Double-checking of each completed interview****:* After the completion of each interview, the enumerators will be required to carefully check all recorded answers, correct any possible errors and conduct re-questioning, if necessary before submission to the central server. Each enumerator has a code tied to each interview in order to be accountable for the quality of the data collection. The supervisor responsible for the survey area will review each completed interview with the check lists and call the attention of the corresponding enumerator for further work, if necessary.

***Provision of survey instruction manual****:* Definition of each question and how to perform the objective interview will be described in detail in the carefully prepared survey instruction manual and the training materials.

***Transmission of data:*** Each enumerator must transmit data according to the timetable agreed upon by his/her field Supervisor. These data will be sent to the secured central server so that the field supervisor and the study team can monitor data collection efforts. In addition, this will protect against data loss, should anything happen to the tablets or smartphones while in the field.

### 3.15 Data Cleaning

After the completion of fieldwork, the data will be exported to Stata statistical software for further cleaning and analysis. The data analyst will perform cleaning and validation of the dataset, detailed labelling of variable and record names to avoid confusion, prepare a detailed codebook with both variable and value labels for all the variables included in the dataset and the “do file” for the cleaning process.

### 3.16 Data Analysis

A more detailed analysis plan will be developed once the survey instrument is finalized. Preliminary analyses will be performed by the authors. Stata statistical analysis software will be used for all data analysis.

Basic probability sampling weights will be calculated for each sampled cluster. Analysis will be carried out to determine the size and characteristics of the target population. We will weight for nonresponse. Descriptive characteristics of the surveyed populations will be provided, using proportions and means. These present the main findings of the study. Cross tabulation of data will be done to assess the interrelationships between two or more variables – notably assessing health expenditures, health care utilization, willingness to pay, and HIV testing across asset wealth quartiles, expenditure groupings, sex, and age.

# 4. Ethical Approval and Human Subjects Considerations

This study will comply with high ethical standards involving human subjects. It is proposed that the following ethical activities shall be accomplished in the context of the study:

a) The study protocol will be submitted to the National Health Research Ethics Committee of Nigeria (NHREC) and Kaduna State Health Research Ethics Committee for ethical approval. We will also submit letters of intent to Kaduna state and local governments informing them of the survey, procedures, and ethical approval obtained for the study. Furthermore, community groups such as youth organizations and traditional leaders will be duly informed to obtain their consent. Before the research team makes contact with potential participants, the study protocol and informed consent forms will be approved in writing by the NHREC. No amendment to the protocol or informed consent process will be implemented without prior Institutional Review Board (IRB) or NHREC approval.

b) All the study personnel will be trained on relevant aspects of good ethical standards that are envisaged in this study. They will also be required to make commitments to abide with the ethical standards.

c) All potential study participants shall be informed about the following:

- The objectives, methods, potential benefits and harm of the research;
- Right to abstain from participation in the study at any time, even during the course of the study; and
- The confidential nature of responses

d) Every individual that participates in the study shall be given adequate information about the study to enable him or her take an informed decision about participation in the study and to formally give or not to give consent to participate. This will be done without pressure or inducement from the study personnel.

### 4.1 Risks

We do not anticipate any serious physical, mental or social risks as a result of participation in this research. Results for all respondents will be presented in aggregate, and no identifiable information will be presented with the study findings.

As with any study, there is a rare chance that the names of study participants become known in the community; however, we feel this poses minimal risk given the nature and topics of the questionnaire.

### 4.2 Benefits

There is no individual benefit for participation in this study. The study results will benefit participating communities and other communities in Kaduna state as the information is used to inform the development of relevant policies and programs to provide financial risk protection options.

### 4.3 Compensation

No compensation will be given to participants in this study.

### 4.4 Informed Consent

Verbal informed consent will be obtained by the enumerators from all participants in the study. Each participant will the consent form read to them and have time to ask questions. It will be stressed that any person may retreat from the study without negative consequences.

### 4.5 Protection of Privacy and Confidentiality

Enumerators will be trained on the importance of privacy and confidentiality, and coached on how to assure potential participants of confidentiality. All researchers will be bound by confidentiality agreements to fully respect the confidentiality of participants. The interview will take place within the household compound. The respondent will be given the opportunity to suggest any conducive place where confidentiality is ensured and distractions are minimized. All individual interviews will be done as privately as possible, with no other relatives, including husbands, able to listen in on the interviews.

Participants’ names will not be recorded anywhere in the final datasets; instead, we will use a unique identification number.

### 4.6 Adverse Events

If any unexpected events or problems occur, interviewers will be trained to immediately contact the study supervisors. Those issues that are protocol violations will be immediately reported to the study team who will be responsible for reporting to the ethics committees and study funders.

All study participants will be given a contact name and telephone number and/or email address to use if they have questions or feel they are encountering problems as a direct result of participating in the study.

# 5. Dissemination of Results

Some data will be used to provide OOP estimates for the State Health Account. Study findings will also be summarized in a final report and shared with the state governments and the Health Finance Technical Working Group. We hope that the results from this study will be used to inform the design and development of financial protection interventions and policies.

# 6. Limitations

This study will only capture a cross section of information from respondents. While cross sectional data can be informative, it does not provide information on changes over time or possible differences due to seasonality. The results are also subject to recall bias due to various recall periods of utilization and expenditure behaviours. Furthermore, the sampling methodology requires consideration of both cost and feasibility which may make results not very generalizable to the entire state population.

1. Kaduna State Ministry of Health Report 2016 [↑](#footnote-ref-1)
2. National Health Accounts 2010-2014 [↑](#footnote-ref-2)
3. The World Bank. Out-of-pocket health expenditure (% of private expenditure on health). Accessed on June 21, 2016, at: <http://data.worldbank.org/indicator/SH.XPD.OOPC.ZS> [↑](#footnote-ref-3)
4. Catastrophic Payments for Health care. Accessed on June 21, 2016 at: <http://siteresources.worldbank.org/INTPAH/Resources/Publications/459843-1195594469249/HealthEquityCh18.pdf> [↑](#footnote-ref-4)
5. Using the recommended urban to rural cluster ratio for Kaduna state by state bureau of statistics. [↑](#footnote-ref-5)
