# Supplementary material for: Factors influencing willingness and ability to pay for social health insurance in Nigeria
Source: PLoS One. 2019 Aug 2;14(8):e0220558. doi: 10.1371/journal.pone.0220558 (PMC6677309; doi:10.1371/journal.pone.0220558)
Supplement: S1 Table — (DOCX) [file pone.0220558.s002.docx]

**Sensitivity Test for Level of Education and Income**

|  | | | | | | | | |
| --- | --- | --- | --- | --- | --- | --- | --- | --- |
| **Output 1:**  **Low= No education _some level of secondary education**  **High= Secondary education completed_ post-secondary education** | |  | B | S.E. | Wald | df | Sig. | Exp(B) |
|  | Income(1) | | .727 | .247 | 8.668 | 1 | .003 | 2.068 |
|  | Sex(1) | | .166 | .505 | .108 | 1 | .743 | 1.180 |
|  | Occupation(1) | | -.719 | .307 | 5.468 | 1 | .019 | .487 |
|  | Education(1) | | -.418 | .265 | 2.502 | 1 | .114 | .658 |
|  | HouseholdSize(1) | | -.442 | .240 | 3.399 | 1 | .065 | .643 |
|  | Locality(1) | | .069 | .249 | .078 | 1 | .780 | 1.072 |
|  | Constant | | 2.207 | .314 | 49.398 | 1 | .000 | 9.089 |
|  | **Output 2**  **Low= No education _some level of primary education**  **High= primary education completed_ post-secondary education** | | B | S.E. | Wald | df | Sig. | Exp(B) |
|  | Income(1) | | .728 | .248 | 8.630 | 1 | .003 | 2.070 |
|  | Sex(1) | | .124 | .502 | .061 | 1 | .804 | 1.132 |
|  | Occupation(1) | | -.836 | .294 | 8.074 | 1 | .004 | .433 |
|  | Education(1) | | -.656 | .548 | 1.432 | 1 | .232 | .519 |
|  | HouseholdSize(1) | | -.375 | .236 | 2.525 | 1 | .112 | .688 |
|  | Locality(1) | | .018 | .245 | .005 | 1 | .941 | 1.018 |
|  | Constant | | 2.183 | .313 | 48.546 | 1 | .000 | 8.870 |
| a. Variable(s) entered on step 1: Income, Sex, Occupation, Education, Household Size, Locality. | | | | | | | | |
